# Association of Serum Galectin-3-Binding Protein and Metabolic Syndrome in a Chinese Adult Population

**DOI:** 10.3389/fendo.2021.726154

**Published:** 2021-11-10

**Authors:** Shihan Zhen, Ruoxin Cai, Xuelian Yang, Yanan Ma, Deliang Wen

**Affiliations:** ^1^ Institute of Health Sciences, China Medical University, Shenyang, China; ^2^ School of Public Health, China Medical University, Shenyang, China

**Keywords:** metabolic syndrome, inflammation, biomarker, sex difference, galectin-3-binding protein

## Abstract

**Background:**

Galectin-3-binding protein (GAL-3BP) is a ubiquitous and multifunctional secreted glycoprotein, which functions in innate immunity and has been highlighted as a potential mediator of adipose inflammation in obesity. In this study, we aimed to identify whether GAL-3BP is a novel biological marker for metabolic syndrome (MetS).

**Methods:**

The biochemical and anthropometric variables of the 570 participants in this study were evaluated using standard procedures. Their serum GAL-3BP levels were measured using enzyme-linked immunosorbent assay (ELISA), while the association between the glycoprotein and MetS was analyzed using multiple logistic regression analyses. Moreover, an experimental MetS model was established. The expression of GAL-3BP in serum and adipose tissue was measured using ELISA and western blotting. Lipid accumulation was determined with the use of immunohistochemistry and immunofluorescent staining.

**Results:**

The serum GAL-3BP level was found to be positively associated with MetS. The logistic regression analyses demonstrated that participants expressing the upper levels of GAL-3BP were more likely to develop MetS than those expressing less of the glycoprotein (OR = 2.39, 95%CI: 1.49, 3.83). The association between the serum GAL-3BP level and MetS was found preferentially in postmenopausal women (OR = 2.30, 95%CI: 1.31, 4.05). In addition, GAL-3BP was increased in the serum and visceral adipose tissue (VAT) of high fat diet (HFD) mice. Moreover, GAL-3BP was highly expressed in VAT macrophages.

**Conclusions:**

This study confirmed serum GAL-3BP to be positively associated with MetS, highlighting it as a useful biological marker of MetS in Chinese participants.

## Introduction

Galectin-3 binding protein (GAL-3BP) is a ubiquitous multifunctional secretory glycoprotein, which was initially identified as having innate immune function in humans following viral and bacterial infections (1). GAL-3BP has several targets, such as Galectin-1, Galectin-3, Galectin-7, Galectin-9, and GAL-3BP, which interact with extracellular matrix proteins and cell surface receptors such as β1-integrins, calcineurin, and NFATc1, thereby regulating cell–cell and cell–matrix interactions (1–5). GAL-3BP is also known to regulate the activation of cyclophilin C, which regulates phagocytosis through the activation of NFAT in macrophages (6). GAL-3BP interacts with a group of target molecules through its multiple functional domains and participates in a wide range of physiological and pathological processes such as cell growth, cellular adhesion, inflammation, and visceral fat increase (1, 4, 7).

Recent studies have suggested that GAL-3BP plays a causal role in innate immunity (1), insulin resistance (8), and chronic low-grade inflammation (9). In humans, GAL-3BP is reported to be elevated in the plasma of obese individuals (10, 11) or those with several symptoms of metabolic syndrome (MetS) (11, 12). In addition, the glycoprotein is secreted from visceral adipose tissues (VAT) (13). In a mouse model, serum GAL-3BP levels increased under a high-fat high-cholesterol diet (14), and several studies have shown that serum GAL-3BP levels can predict the severity of liver disease, especially non-alcoholic fatty liver disease (NAFLD) (7, 14).

MetS refers to the cluster of biological factors that feature in type 2 diabetes mellitus, hypertension, dyslipidemia, and abdominal obesity (15) and is becoming a major public health issue (16). With the increase in obesity, the incidence of MetS in the Chinese population has increased rapidly from 29.65% in 2005 to 45.49% in 2014 (17). This increase also elevates the incidence of arthritis, diabetes, and cardiovascular disease (18, 19). Therefore, it is vital to examine the potential mechanisms that underlie MetS and identify biomarkers that will help to assess the risk of developing the syndrome.

Although the relationship between GAL-3BP and human obesity has been demonstrated (11), the significance of GAL-3BP as a biomarker for MetS has not been fully examined to date. The aims of the study were to investigate the clinical significance of serum GAL-3BP levels in determining the complex phenotype of MetS and evaluate whether GAL-3BP can act as a suitable biomarker for MetS by assessing the correlation between them.

## Materials and Methods

### Human Subjects

The study was based on the Major Chronic Diseases Prevention and Control Cohort in Northeast China, a well-designed prospective cohort used to investigate environmental and genetic factors in non-communicable chronic disease. A face-to-face interview was conducted to collect information using a standardized questionnaire. The eligibility of the participants was defined as those who had resided in the area for at least 5 years, could partake in barrier-free communication, were compliant, and were 1) free from severe physical disabilities, cancer, cerebrovascular disease, severe liver and kidney diseases or psychological disorders or dementia over the past 6 months; 2) not currently diagnosed with a communicable disease; 3) not pregnant. All participants provided written informed consent. Between September and December 2019, a total of 675 participants from Yuhong district were enrolled using a multistage sampling technique. Sixty-nine participants were excluding because of hemolysis or chylous blood (fatty blood). Of the remaining 606 participants, 36 completed only the short questionnaire. After exclusions, the data for 570 participants were made available for the current investigation. Participants provided written informed consent to undergo venipuncture and were all told of the intended use of the samples. The research was approved by the Ethics Committee of China Medical University (CMU).

Waist circumference (WC) was measured at umbilicus level in the standing position. Systolic (SBP) and diastolic (DBP) blood pressure readings were taken using an automatic electronic sphygmomanometer (HEM-907; Omron, Tokyo, Japan). Blood was collected from the anterior humerus vein in the morning after 12-h of fasting without the intake of medication. Fasting plasma glucose (FPG), triglyceride (TG), low- and high-density lipoprotein-cholesterol (LDL- and HDL-C), aspartate aminotransferase (AST) and alanine aminotransferase (ALT) levels were determined using standard procedures.

### Definition of Metabolic Syndrome

Participants with MetS were defined according to the criteria set out by the International Diabetes Federation (20). MetS was diagnosed when subjects presented with abdominal obesity (defined as WC ≥ 90 cm for males or ≥80 cm for females) and two or more of the following criteria: 1) high blood pressure (SBP ≥ 130mmHg or DBP ≥ 85mmHg); 2) elevated plasma glucose (FPG ≥ 5.6mmol/L); 3) elevated TG (TG ≥ 1.7mmol/L); or 4) low HDL-C (HDL-C < 1.04mmol/L for males or < 1.3mmol/L for females).

### Covariates

Covariates including age, gender, nationality (Han or other), educational attainment (illiterate or primary school; junior middle school; high middle school; and college or higher), menopausal status (pre- or post-menopausal), AST (U/L), and ALT (U/L) were collected using face-to-face interviews and general information questionnaires.

### Animals

C57BL/6 mice weighing approximately 20 g at the beginning of the experimental procedure were used. Mice were housed in a 12 h/12 h light/dark cycle and given distilled water and feed. Mice over 12–16 weeks-of-age were randomly divided into two groups. The control diet (CD) group was provided with a standard CD. The high fat diet (HFD) group was fed on a diet in which 60% of the calories were obtained from fat (Research diet #D12492) for 12 weeks. MetS was induced by feeding the animals with a HFD (Research Diets, New Brunswick, NJ), which was consistent with previously published work (21). Blood samples were used to measure blood glucose, lipoprotein, and TG levels. Food intake, water consumption, weight, and body compositions were measured weekly, and the average food and water consumption was calculated accordingly. We used gonadal white adipose tissue for measurements. Before tissue collection, the mice fasted for 16 h and were weighed, before samples were excised and fixed in 4% paraformaldehyde buffer for histopathological assessment. Serum and various tissue samples were collected and frozen at −80°C. All animal procedures were approved by the Animal Ethics Committee of China Medical University.

### Measurements of GAL-3BP

Human Galectin-3BP ELISA (2H-KMLJh314728) and Mouse Galectin-3BP ELISA (2M-KMLJM228552m) kits were purchased from CAMILO biological (Nanjing, China). Both kits were used in accordance with the manufacturer’s instructions. Standards provided with the kits were diluted to produce a gradient of biomarker concentrations to obtain standard curves.

### Western Blots

Visceral adipose tissues were prepared and lysed according to standard protocols. Antibodies to G3BP (ab181150, 1:1000) and GAPDH (ab8245, 1:2000) were purchased from Abcam. Blotting membranes were incubated with the primary antibody at 4°C overnight and the secondary anti-rabbit IgG (#32731; 1:10000; Thermo Scientific) at room temperature for 1 h. The resulting bands were visualized using a Tanon 5500 imaging system (Tanon, Shanghai, China). The results were quantified using ImageJ software (National Institute of Mental Health, USA).

### Immunohistochemistry

Tissues were fixed overnight in 4% paraformaldehyde in PBS, dehydrated in a graded ethanol series, and washed with xylene. Tissues were embedded in paraffin and sectioned as 5 µm. Single-label immunohistochemistry was performed on adipose tissues. Macrophages were detected using a monoclonal antibody against F4/80 (ab6640, 1:100). Histopathological images were captured by immunofluorescence microscopy (80I, Nikon Corporation, Tokyo, Japan). Three sections per mouse were analyzed and n = 11–20.

### Immunofluorescence Staining

After being deparaffinized in xylene and rehydrated using an ascending ethanol series, the slides were permeabilized with 0.1% Triton-X 100 for 5 min, blocked with 10% goat serum in PBST (PBS with 0.05% Tween 20) for 1 h at 37°C, and incubated with GAL-3BP (1:100) and F4/80 (1:100) at 4°C overnight. After washing with PBST, the coverslips were mounted with anti-fade reagent and 4′, 6′-diamidino-2-phenylindole (DAPI) (Life Technologies, Waltham, MA, USA). Images were acquired using a Leica DFC310 FX digital camera connected to a Leica DMI4000 B light microscope (Wetzlar, Germany).

### Statistical Analysis

Descriptive information was presented is means with standard deviations. ANOVA tests for continuous variables and chi-square tests for categorical variables were used to compare participants with and without MetS. GAL-3BP concentration was divided into tertiles, and an increase from T1 to T3 was assumed (22). The upper strata of GAL-3BP levels were defined as T2 and T3, whereas and lower strata were defined as T1. The cut-off value in the present study were 45.13 (ng/ml).

Logistic regression models were used to estimate the odds ratios (OR) and 95% confidence intervals (95%CI) for MetS and serum GAL-3BP levels. The age-adjusted model was adjusted for age, and the multiple-adjusted model was adjusted for gender, national, educational attainment, AST, and ALT. A *P*-value < 0.05 indicated statistical significance. Statistical analyses were carried out using SPSS 25.0 (SPSS, Inc., Chicago, IL) and Stata 13.0 (StataCorp, College Station, TX, USA), while R (R studio, USA) and Graphpad Prism 5.0 (GraphPad Inc., La Jolla, CA) were adopted for graph preparation.

## Results

**Table 1** shows the characteristics of the participants; 25.09% of participants had MetS. Compared with subjects without MetS, those with MetS exhibited higher values for WC, BMI, blood pressure, FPG, TG, HDL-C, LDL-C, and GAL-3BP. The median overall GAL-3BP concentration was 70.55 ng/ml (SD = 52.12, SEM = 2.18). The median GAL-3BP concentration for participants with MetS was 80.31 ng/ml (SD = 68.21, SEM = 5.70), whereas that for participants without MetS was 67.28 ng/ml (SD = 45.10, SEM = 2.18). The median GAL-3BP concentration in females was 72.20 ng/ml (SD = 55.35, SEM = 2.80), whereas that in males was 66.95 ng/ml (SD = 44.17, SEM = 3.30). Further, the median GAL-3BP concentration in females with MetS was 80.77 ng/ml (SD = 69.74, SEM = 6.56), whereas that in females without MetS was 68.71 ng/ml (SD = 48.00, SEM = 2.88) (*p <*0.05). The median GAL-3BP concentration in males with MetS was 78.59 ng/ml (SD = 63.19, SEM = 11.54), whereas that in males without MetS was 64.61 ng/ml (SD = 39.13, SEM = 3.21) (*p >*0.05). In addition, Gal-3BP levels in females and males were shown in **Supplemental Table 1**.

**Table 1:** Characteristics of study participants with and without MetS.

Characteristic	Participants without MetS (n = 427)	Participants with MetS (n = 143)	*P* value
Age, Mean (SD)	60.74 (9.95)	64.92 (7.48)	**<0.001**
Female, No. (%)	278 (65.11)	113 (79.02)	**0.002**
National (Han), No. (%)	368 (86.18)	130 (90.91)	0.141
Educational attainment, No. (%)			0.238
Illiterate or primary school	97 (22.72)	38 (26.57)	
Junior middle school	165 (38.64)	49 (34.27)	
High middle school	134 (31.38)	51 (35.66)	
College or higher	31 (7.26)	5 (3.5)	
Anthropometry			
WC, (cm), Mean. (SD)	78.84 (8.09)	89.04 (6.76)	**<0.001**
BMI, (kg/m2), Mean. (SD)	24.67 (2.92)	27.71 (3.13)	**<0.001**
SBP, (mmHg), Mean. (SD)	126.90 (14.47)	135.59 (11.81)	**<0.001**
DBP, (mmHg), Mean. (SD)	75.31 (8.80)	77.91 (9.33)	**0.003**
Laboratory examinations			
FPG, (mmol/L), Mean. (SD)	5.34 (1.21)	6.17 (1.78)	**<0.001**
TG, (mmol/L), Mean. (SD)	1.55 (1.33)	2.34 (1.36)	**<0.001**
HDL-C, (mmol/L), Mean. (SD)	1.31 (0.29)	1.22 (0.34)	**0.003**
LDL-C, (mmol/L), Mean. (SD)	3.25 (0.76)	3.45 (0.77)	**0.006**
AST, (U/L), Mean (SD)	26.36 (10.04)	26.22 (9.87)	0.882
ALT, (U/L), Mean (SD)	24.67 (17.75)	26.76 (13.47)	0.200
G3BP, (ng/ml), Mean (SD)	67.28 (45.10)	80.31 (68.21)	0.001

P-values < 0.05 are bold.

### The Association Between GAL-3BP and MetS

After adjusting for confounders, participants with the higher GAL-3BP levels showed higher odds of having MetS than those in with lower levels of GAL-3BP (OR = 2.39, 95%CI: 1.49, 3.83). To further define any sex differences, we compared the results obtained for male and female subjects. Females with higher levels of GAL-3BP had higher odds of having MetS than those with lower levels of GAL-3BP (OR=2.31, 95%CI: 1.35, 3.94), whereas, males with higher GAL-3BP levels also showed higher odds of having MetS than those with lower levels of GAL-3BP (OR=3.02, 95%CI: 1.03, 8.34). Further, females were divided into premenopausal and postmenopausal groups. In the postmenopausal group, participants with higher GAL-3BP levels had a higher incidence of MetS than those with lower levels of the glycoprotein (OR=2.30, 95%CI: 1.31, 4.05). These results are shown in **Tables 2**, **3**. In addition, the association between GAL-3BP levels and MetS components were shown in **Supplemental Table 2**.

**Table 2:** Association between Gal-3BP level and MetS.

	Level of GAL-3BP		*P* value
	Lower	Upper	
Total (n=570)			
Age-adjusted model	1 (Reference)	**2.40 (1.52, 3.80)**	**<0.001**
Multiple-adjusted model	1 (Reference)	**2.39 (1.49, 3.83)**	**<0.001**
Male (n=179)			
Age-adjusted model	1 (Reference)	**3.03 (1.09, 8.39)**	**0.033**
Multiple-adjusted model^a^	1 (Reference)	**3.02 (1.03, 8.34)**	**0.043**
Female (n=391)			
Age-adjusted model	1 (Reference)	**2.32 (1.37, 3.93)**	**0.002**
Multiple-adjusted model^b^	1 (Reference)	**2.31 (1.35, 3.94)**	**0.002**

OR, Odds ratio; CI, confidence interval; Age-adjusted model, adjusted for age (in years); Multiple-adjusted model, additional adjusted for gender, national, educational attainment, AST(U/L) and ALT(U/L). ^a^Multiple-adjusted model adjusted for age (in years), national, educational attainment, AST(U/L) and ALT(U/L). ^b^Multiple-adjusted model adjusted for age (in years), national, educational attainment, menopausal, AST(U/L) and ALT(U/L). P-values < 0.05 are bold.

**Table 3:** Association between Gal-3BP level and MetS in female subjects.

	Level of GAL-3BP		*P* value
	Lower	Upper	
Premenopausal (n=63)			
Age-adjusted model	1 (Reference)	1.09 (0.19, 6.28)	0.927
Multiple-adjusted model	1 (Reference)	1.24 (0.17, 9.10)	0.833
Postmenopausal (n=328)			
Age-adjusted model	1 (Reference)	**2.42 (1.39, 4.22)**	**0.002**
Multiple-adjusted model	1 (Reference)	**2.30 (1.31, 4.05)**	**0.004**

OR, Odds ratio; CI, confidence interval; Age-adjusted model, adjusted for age (in years); Multiple-adjusted model, additional adjusted for national, educational attainment, AST(U/L) and ALT(U/L). P-values < 0.05 are bold.

### GAL-3BP Was Increased in the Serum and VAT of Mice on a High-Fat Diet

**Figure 1:**
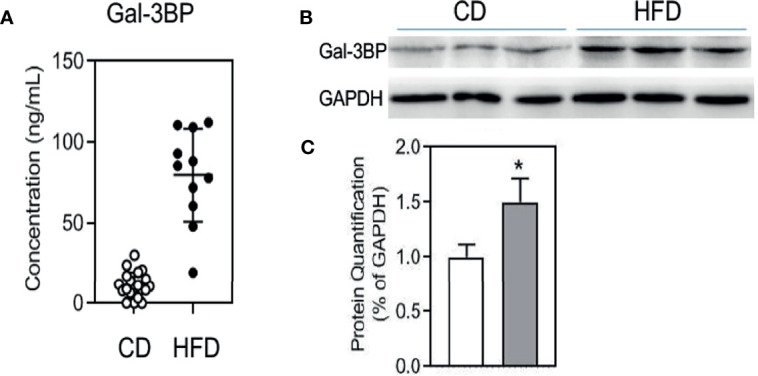
GAL-3BP demonstrated positive correlations with MetS. **(A)** Serum GAL-3BP concentration. **(B)** Protein expressions of GAL-3BP and internal control GAPDH in Visceral Adipose Tissues. **(C)** Quantification of proteins with normalization to protein levels of GAPDH. Data are represented as mean ± SEM. n = 11-20, *p < 0.05 *vs* CD mice.

To confirm the GAL-3BP expression pattern in MetS mice, we fed the mice with a HFD. Data on the assessment of MetS parameter in mice were shown in **Supplemental Table 3**. As shown in **Figure 1A**, GAL-3BP was found to be increased significantly in the serum of the HFD group compared with that in the control group. We then assessed whether GAL-3BP was secreted from VAT and found that levels were increased in the HFD group compared with the control group (**Figures 1B, C**).

### GAL-3BP Was Highly Expressed in VAT Macrophages

**Figure 2:**
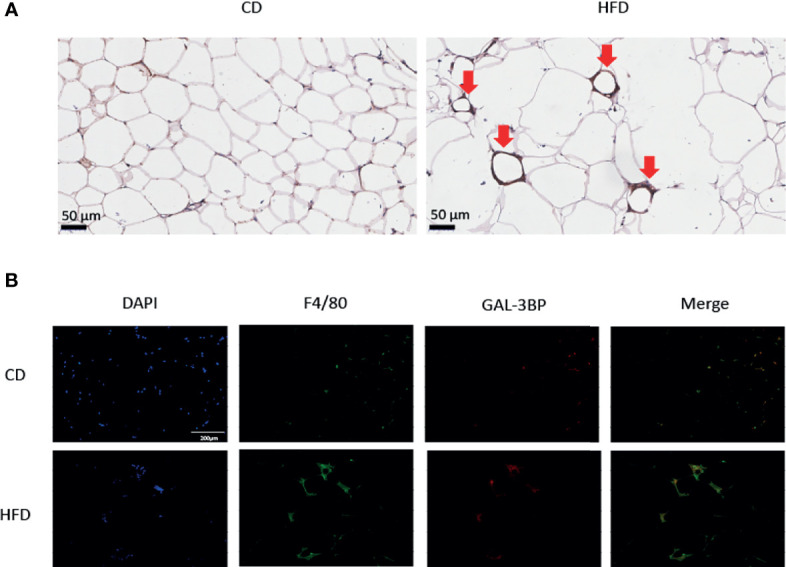
The expression of GAL-3BP in the Visceral Adipose Tissues. **(A)** Representative images of F4/80 staining in VAT after HFD or CD exposure. 100 × **(B)** Representative images of immunofluorescent staining for F4/80 and GAL-3BP adducts. 100 ×. n = 5.

To verify the VAT cell types that secreted GAL-3BP, we tested the expression of the glycoprotein in the IHC. As shown in **Figure 2A**, in HFD group VAT, the adipocytes were bigger in the HFD group than the CD group. GAL-3BP was highly expressed as in a crown shape, which indicated that the macrophages may secrete the protein in the VAT of the HFD group. Next, using the macrophage marker F4/80 to locate the cells, we found that GAL-3BP was expressed in cells identified by F4/80 (**Figure 2B**). In the VAT of the HFD group, GAL-3BP and F4/80 were more highly expressed than in the CD group (**Figure 2B**).

## Discussion

In this study, we demonstrated that the serum GAL-3BP levels were positively correlated with the incidence of MetS in humans, particularly in postmenopausal females. Serum GAL-3BP may therefore serve as a useful biological marker for MetS. GAL-3BP was highly expressed in the VAT of MetS mice, suggesting that GAL-3BP expression may represent a biological process that underlies MetS. Moreover, we newly identified adipose tissue macrophages as a source of Gal3-BP under conditions of adipose tissue expansion.

In this study, we were able to demonstrate the usefulness of the serum GAL-3BP levels in reflecting the incidence of MetS. Associations between serum GAL-3BP, abdominal obesity and lipoprotein levels have been shown previously (9–11), and we also identified associations between GAL-3BP level, center obesity and high TG, while further demonstrating the positive association between GAL-3BP and MetS. In a previous report, GAL-3BP, a large oligomeric glycoprotein, was suggested to be the galectin-3 ligand (23). Previous studies have indicated that the level of galectin-3 is associated with visceral fat, lipoprotein levels, glucose homeostasis, and even the presence of MetS (24, 25). Therefore, GAL-3BP may affect the distribution of body fat, gluconeogenesis, hyperglycemia, and lipolysis, which may result in the reduced ability to maintain metabolic homeostasis.

Logistic regression analyses suggested that GAL-3BP is positively correlated with MetS. The sex difference may be due to the sexual dimorphisms in adipose tissue biology, including adipose distribution and function (26–28). Cai et al. reported GAL-3BP increase in NAFLD patients between three groups (PostM-NAFLD vs. PostM-Control, PreM-NAFLD vs. PreM-Control, and PostM-NAFLD vs. PreM-NAFLD). They hypothesized GAL-3BP may connect to NALFD and metabolic disorders (7). Our results also report the association between GAL-3BP and MetS in postmenopausal women. Subanalyses of females further suggested the presence of a robust association between GAL-3BP and MetS in postmenopausal females. Although the mechanism underlying menopausal status and an association between serum GAL-3BP and MetS is unclear, several studies have suggested that biological changes after menopause may lead to the reduced ability of adipose tissue to expand, leading to additional fat storage (27, 28). The change in adipose tissue expandability may underlie the significant association between serum GAL-3BP and MetS in postmenopausal women. In addition, GAL-3BP was shown to interact with Complement Factor D, Insulin like Growth Factor 1, and Albumin directly, and to network with Estrogen Receptor 1 (ESR1), Nitric Oxide Synthase 3 and INS (7). Several studies have suggested that estrogen is associated with MetS and its related factors in postmenopausal women (29, 30). In premenopausal women, intact estrogen dependency might be preserved in the myometrium, as well as in the uterine endometrium with characteristic stable expression of ESR1 with ESR2, whereas, in postmenopausal women with much lower estrogen levels, ESR1 is decreased (31). An imbalance between ESR1 in the adipose tissue could therefore affect the development of metabolic diseases (32). Hormones have critical functions in MetS pathogenesis and progression, and estrogens have critical functions in lipoprotein metabolism. Reduced estrogen in postmenopausal women may enhance the association between the Gal-3BP and MetS. The association between GAL-3BP and ESR1 may thus result in postmenopausal MetS.

GAL-3BP was shown to be highly expressed in the VAT, which is consistent with previous findings (10, 11). Roelofsen et al. reported that GAL-3BP was secreted from VAT (13). We also found that serum GAL-3BP was highly expressed in the VAT of MetS mice. Typically, the increase in visceral fat has been verified to further boost insulin resistance, while MetS is probably induced by insulin resistance caused by the association between GAL-3BP levels and visceral adiposity. Moreover, recent data from reconstituted proteins *in vitro* have confirmed the association between GAL-3BP and adiponectin. GAL-3BP is a novel serum adiponectin binding protein and may abrogate the anti-inflammatory effects of adiponectin (9, 11). Hypoadiponectinemia is closely associated with hypertension, dyslipidemia, diabetes mellitus, and visceral fat obesity related to MetS (33). The present study showed that GAL-3BP was highly expressed in VAT macrophages. Inflammation may also account for these associations. Previous data have indicated that Gal‐3BP has immunosuppressive as well as immunostimulatory functions *in vitro* (1); the glycoprotein has been implicated in inflammatory distress, immune response (7), and chronic low-grade inflammation (9). GAL-3BP is significantly positively associated with inflammatory markers, including IL6, IL-1β, together with TNFα (11, 34, 35), and these may participate in MetS pathogenesis related to GAL-3BP, since inflammation may probably result in insulin resistance. Furthermore, Gleissner et al. reported that GAL-3BP induces a pro-inflammatory transcriptome in human monocyte-derived macrophages (11). This is in line with our finding that GAL-3BP was highly expressed in macrophages in adipose tissues. Simultaneously, several studies have reported that GAL-3BP is a new biomarker for predicting chronic pancreatitis, non-alcoholic steatohepatitis (NASH), and NAFLD (7, 34, 36). Elevated GAL-3BP in patients with MetS is consistent with the presence of chronic low-grade inflammation as a key characteristic of MetS, pancreatitis and NASH (9, 34). We may compare GAL-3BP with inflammatory parameters in future.

Certain limitations to this study should be noted. First, this was a cross-sectional study to determine the significance of serum GAL-3BP as a biological marker of MetS. The cross-sectional design limited the usefulness of evaluating the serum GAL-3BP level as a biomarker in predicting the progression of MetS. A prospective study based on baseline stratified serum GAL-3BP levels may be necessary. Second, our study does not clearly demonstrate a mechanism underlying the association between GAL-3BP and MetS in participants. The murine model did not fully explain the source of Gal3-BP under adipose tissue expansion conditions in human subjects. We did not obtain adipose tissues from participants, who were community residents. We may delve into the mechanisms and try to link the findings in the future. Third, we did not include a perimenopausal group with oligomenorrea and perimenopausal symptoms. Participants going through menopausal process (perimenopausal women) were probably self-classified as menopausal. As menopause is a gradual process, the perimenopausal period should be included in future studies. Finally, we did not assess other adipokine (adiponectin or leptin, etc.) or other biomarkers levels, it is difficult to compare the GAL-3BP with other biomarkers. Therefore, we may compare GAL-3BP with adiponectins and other biomarkers in terms of the receiver operating characteristics for MetS in future.

Our results suggest a significant role for GAL-3BP in reflecting the complex phenotypes of MetS. In conclusion, the results from this study demonstrate that GAL-3BP levels show positive associations with MetS. This finding is particularly important because of the increasing risk of MetS seen in the Chinese population. Understanding the role of GAL-3BP in altering MetS could assist the development of diagnostic tools and treatments for obesity-related metabolic disorders. Nonetheless, further studies should be carried out to clarify the role of GAL-3BP as a biomarker for MetS.

## Data Availability Statement

Data are available upon reasonable request. Requests to access the datasets should be directed to DW, dlwen@cmu.edu.cn.

## Ethics Statement

The studies involving human participants were reviewed and approved by China Medical University. The patients/participants provided their written informed consent to participate in this study. The animal study was reviewed and approved by China Medical University.

## Author Contributions

YM and DW conceived and designed the study. SZ, RC, and XY collected, managed, and analyzed the data. SZ drafted the manuscript. All authors contributed to the article and approved the submitted version.

## Funding

This research was supported by the National Key R&D Program of China (Grant #2018YFC1311600) and Liaoning Revitalization Talents Program (Grant #XLYC1808036).

## Conflict of Interest

The authors declare that the research was conducted in the absence of any commercial or financial relationships that could be construed as a potential conflict of interest.

## Publisher’s Note

All claims expressed in this article are solely those of the authors and do not necessarily represent those of their affiliated organizations, or those of the publisher, the editors and the reviewers. Any product that may be evaluated in this article, or claim that may be made by its manufacturer, is not guaranteed or endorsed by the publisher.

## Abbreviations

GAL-3BP, Galectin-3-binding protein; MetS, metabolic syndrome; WC, waist circumference; SBP, systolic blood pressure; DBP, diastolic blood pressure; FPG, fasting plasma glucose; TG, triglycerides; HDL-C, high-density lipoprotein-cholesterol; LDL-C, low-density lipoprotein-cholesterol; AST, aspartate aminotransferase; ALT, alanine aminotransferase; ECM, extracellular matrix; ELISA, enzyme linked immunosorbent assay; VAT, visceral adipose tissue; CD, Control diet; HFD, High fat diet.
