# Supplementary material for: Association of Serum Galectin-3-Binding Protein and Metabolic Syndrome in a Chinese Adult Population
Source: Front Endocrinol (Lausanne). 2021 Nov 10;12:726154. doi: 10.3389/fendo.2021.726154 (PMC8631730; doi:10.3389/fendo.2021.726154)
Supplement: Supplementary file 1 [file Table_1.docx]

Supplemental Table 1. Gal-3BP levels in females and males.

| Characteristic | Male | Female | *P* value |
| --- | --- | --- | --- |
| G3BP levels, No. (%) |  |  | 0.655 |
| Upper | 117 (65.36) | 263 (67.26) |  |
| Lower | 62 (34.64) | 128 (32.74) |  |

Supplementary Table 2. Association between Gal-3BP level and MetS component

|  | Level of GAL-3BP | | *P* value |
| --- | --- | --- | --- |
|  | Lower | Upper |  |
| Center obesity |  |  |  |
| Age-adjusted model | 1 (Reference) | **1.70 (1.18, 2.45)** | **0.005** |
| Multiple-adjusted model | 1 (Reference) | **1.71 (1.16, 2.51)** | **0.007** |
| Hypertension |  |  |  |
| Age-adjusted model | 1 (Reference) | 1.30 (0.91, 1.86) | 0.155 |
| Multiple-adjusted model | 1 (Reference) | 1.31 (0.91, 1.88) | 0.150 |
| hyperglycemia |  |  |  |
| Age-adjusted model | 1 (Reference) | 1.02 (0.70, 1.51) | 0.905 |
| Multiple-adjusted model | 1 (Reference) | 1.02 (0.68, 1.52) | 0.927 |
| High TG |  |  |  |
| Age-adjusted model | 1 (Reference) | 1.41 (0.97, 2.04) | 0.069 |
| Multiple-adjusted model | 1 (Reference) | **1.47 (1.00, 2.16)** | **0.048** |
| Low HDL-C |  |  |  |
| Age-adjusted model | 1 (Reference) | 1.10 (0.77, 1.56) | 0.601 |
| Multiple-adjusted model | 1 (Reference) | 1.07 (0.74, 1.55) | 0.705 |

Note: OR: Odds ratio, CI: confidence interval; Age-adjusted model: adjusted for age (in years); Multiple-adjusted model: additional adjusted for gender, national, educational attainment, AST(U/L) and ALT(U/L). *P*-values< 0.05 are bold.

Supplementary Table 3. Characteristics of mice

|  | CD | HFD | *P* value |
| --- | --- | --- | --- |
| Body Weight, (g), Mean. (SD) | 26.1 (2.22) | 45.37 (3.81) | **<0.001** |
| FPG, (mmol/L), Mean. (SD) | 55.18 (9.82) | 100.92 (25.31) | **<0.001** |
| Gonadal adipose tissue mass (% of BW), Mean. (SD) | 1.86 (0.64) | 13.44 (3.36) | **<0.001** |
| TG, (mmol/L), Mean. (SD) | 0.69 (0.14) | 0.97 (0.15) | **<0.001** |
| LDL, (mmol/L), Mean. (SD) | 0.90 (0.24) | 1.84 (0.28) | **<0.001** |
| HDL, (mmol/L), Mean. (SD) | 1.47 (0.16) | 1.28 (0.19) | **0.019** |

*P*-values< 0.05 are bold.
